# Supplementary material for: Monitoring the microbiome for food safety and quality using deep shotgun sequencing
Source: NPJ Sci Food. 2021 Feb 8;5:3. doi: 10.1038/s41538-020-00083-y (PMC7870667; doi:10.1038/s41538-020-00083-y)
Supplement: Supplementary file 1 — Supplemental Figures [file 41538_2020_83_MOESM1_ESM.pdf]

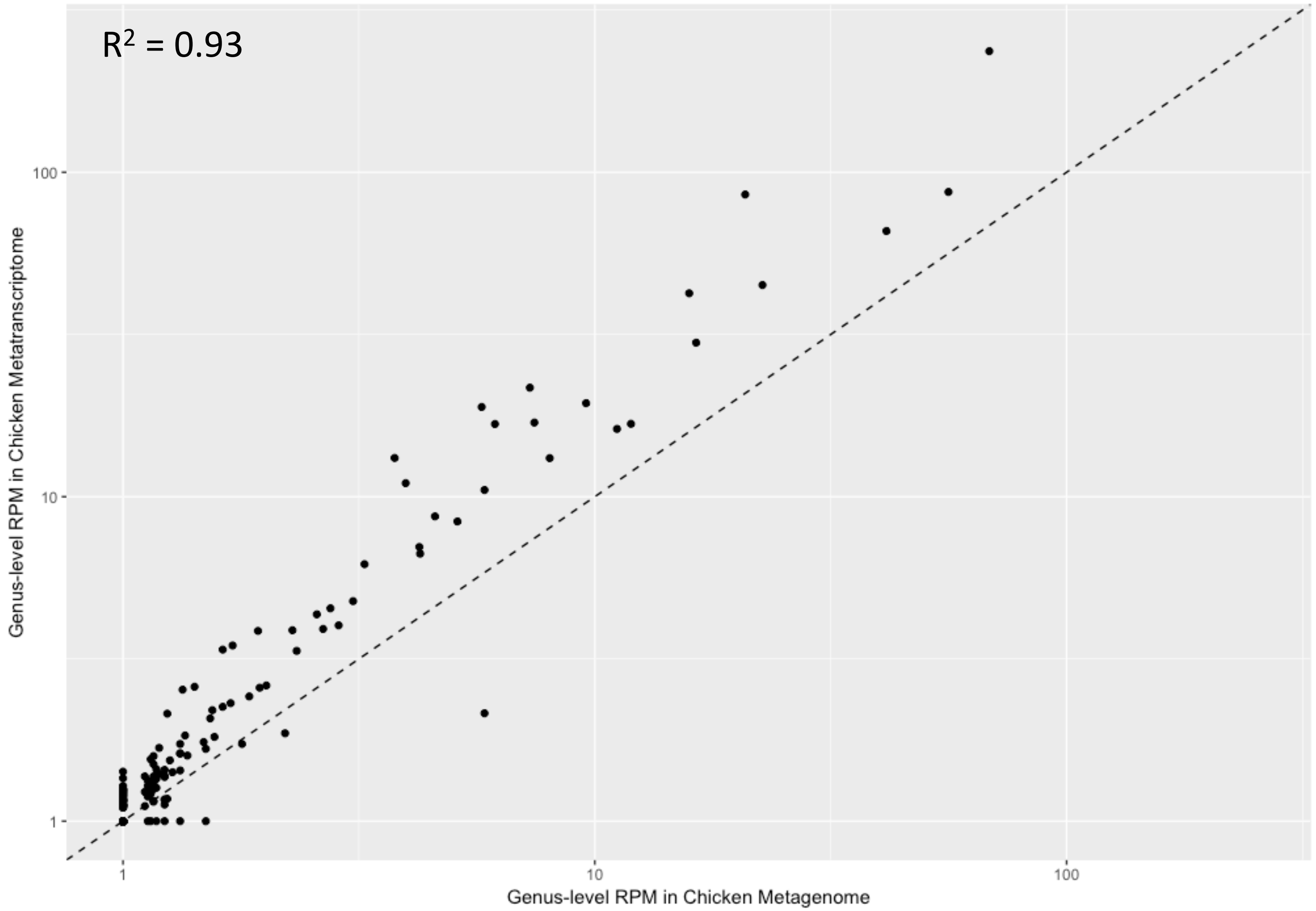

Supplementary Figure 1: Genus-level relative abundance (RPM) in total RNA metatranscriptome and total DNA metagenome high protein powder samples of the same starting material.

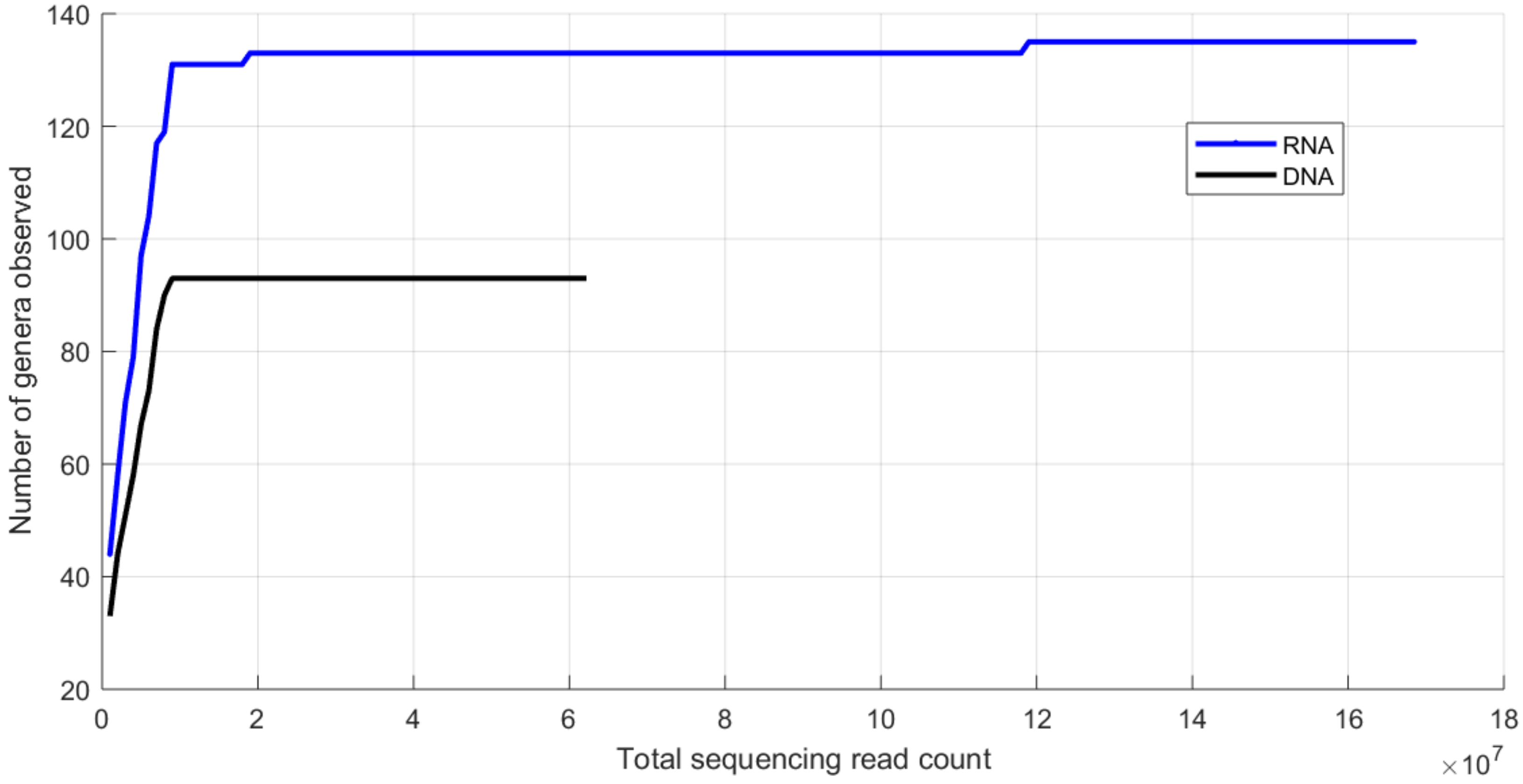

Supplementary Figure 2: Alpha diversity (number of genera) for total RNA metatranscriptome and total DNA metagenome sequencing of high protein powder samples of the same starting material, for a range of *in silico* subsampled sequencing depths (total sequence read counts including food matrix sequences).

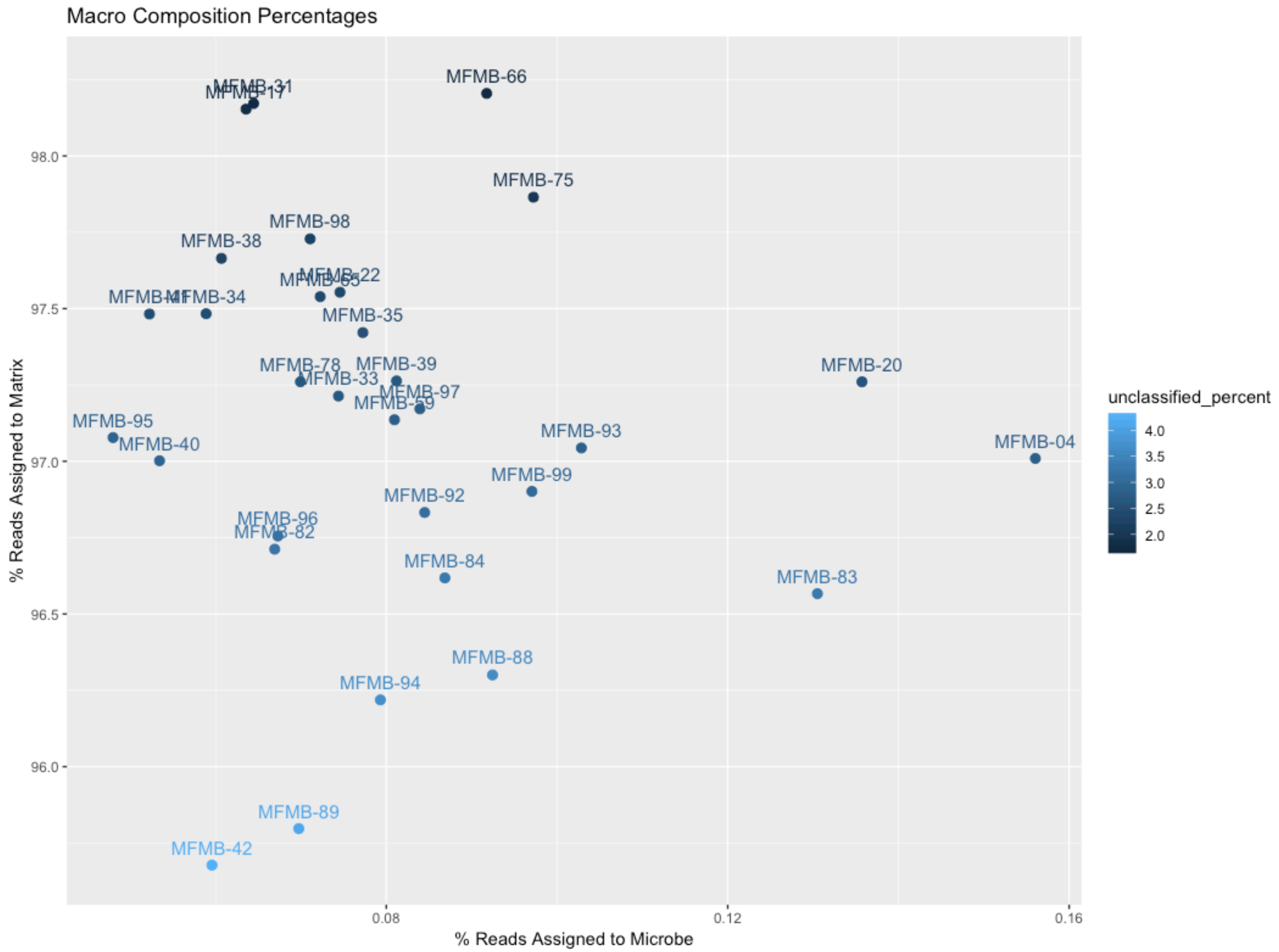

Supplementary Figure 3: Macro-composition variations from total RNA metatranscriptome sequencing for each high protein powder sample (n=31): the percentage of reads passing quality control that were assigned to matrix, microbes, or could not be assigned (unclassified).

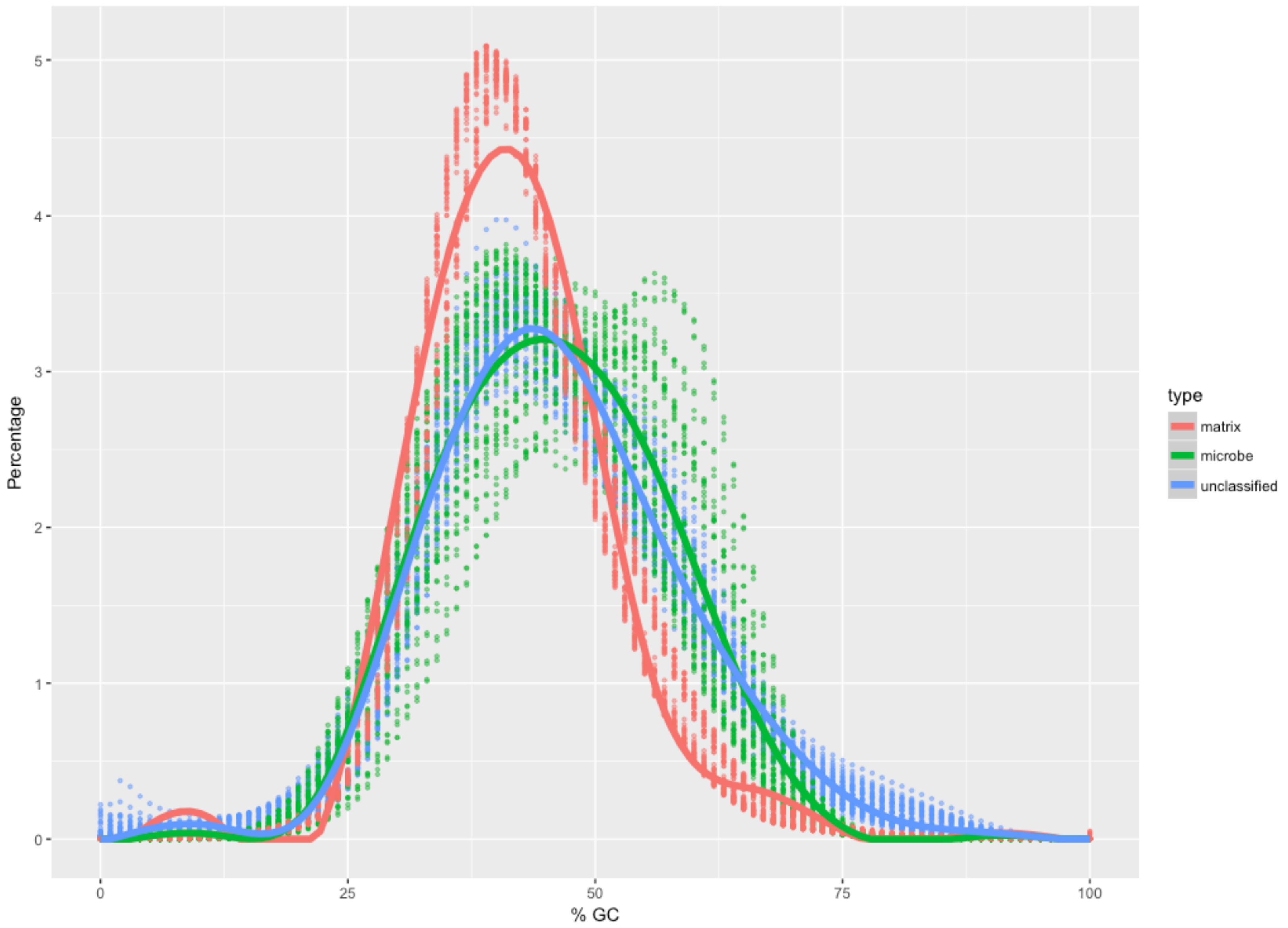

Supplementary Figure 4: GC content of reads for three macro-composition origination groups: matrix (red), microbe (green) and unclassified (blue) for all samples (n=31) as points, with a trend line shown as a solid line using a generalized additive model.

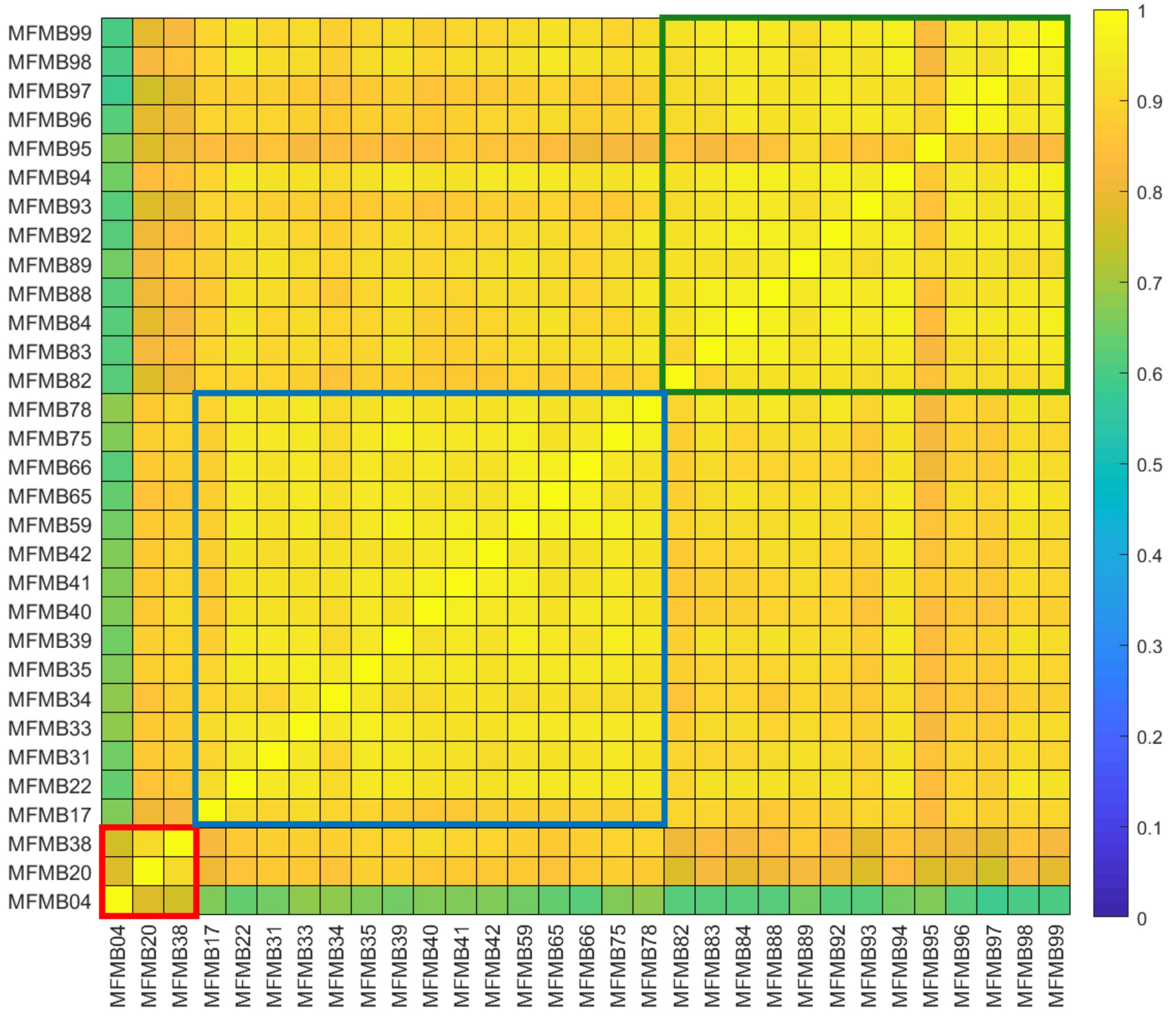

Supplementary Figure 5: Pairwise Spearman correlation of the samples' RPM values of detected 229 genera. The matrix contaminated samples (from Supplier A) are indicated by a red rectangle, while the remaining Supplier A samples with a blue rectangle, and the Supplier B samples with a green rectangle.

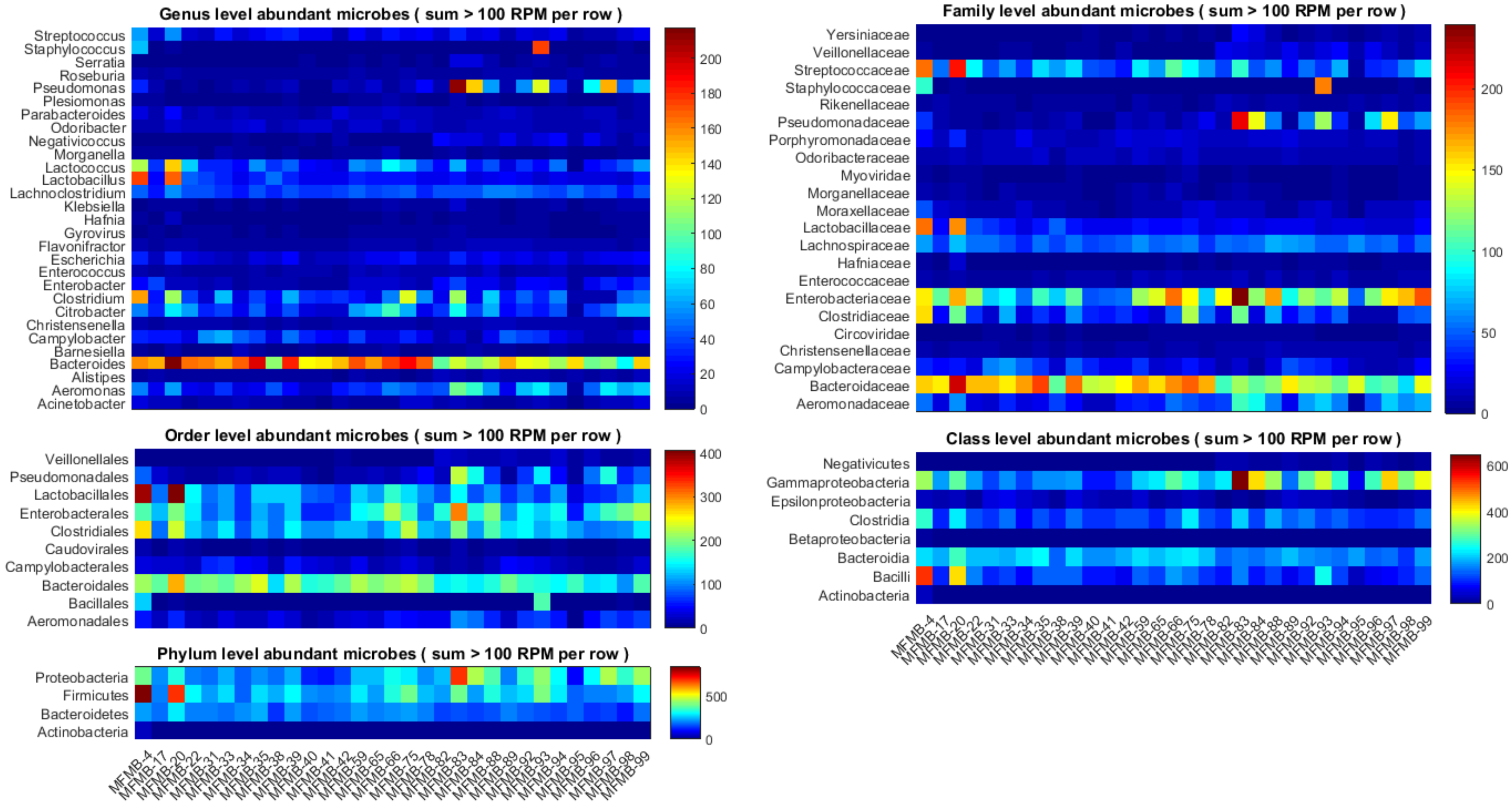

Supplementary Figure 6: Most abundant microbes at various taxonomic levels, including only those with a sum of RPM > 100 across all samples.
